# Recent advances in the potential effects of natural products from traditional Chinese medicine against respiratory diseases targeting ferroptosis

**DOI:** 10.1186/s13020-024-00918-w

**Published:** 2024-03-22

**Authors:** Tian Chen, Lu Ding, Meiru Zhao, Siyu Song, Juan Hou, Xueyan Li, Min Li, Kai Yin, Xiangyan Li, Zeyu Wang

**Affiliations:** 1 College of Integrated Traditional Chinese and Western Medicine, Changchun University of Chinese Medicine, Changchun, China; 2 Northeast Asia Research Institute of Traditional Chinese Medicine, Key Laboratory of Active Substances and Biological Mechanisms of Ginseng Efficacy, Ministry of Education, Jilin Provincial Key Laboratory of Bio-Macromolecules of Chinese Medicine, Changchun University of Chinese Medicine, Changchun, Jilin, 130117 China; 3 Research Center of Traditional Chinese Medicine, College of Traditional Chinese Medicine, Changchun University of Chinese Medicine, Changchun, Jilin, 130021 China

**Keywords:** TCM, Natural products, Ferroptosis, Respiratory diseases, Mechanism

## Abstract

Respiratory diseases, marked by structural changes in the airways and lung tissues, can lead to reduced respiratory function and, in severe cases, respiratory failure. The side effects of current treatments, such as hormone therapy, drugs, and radiotherapy, highlight the need for new therapeutic strategies. Traditional Chinese Medicine (TCM) offers a promising alternative, leveraging its ability to target multiple pathways and mechanisms. Active compounds from Chinese herbs and other natural sources exhibit anti-inflammatory, antioxidant, antitumor, and immunomodulatory effects, making them valuable in preventing and treating respiratory conditions. Ferroptosis, a unique form of programmed cell death (PCD) distinct from apoptosis, necrosis, and others, has emerged as a key area of interest. However, comprehensive reviews on how natural products influence ferroptosis in respiratory diseases are lacking. This review will explore the therapeutic potential and mechanisms of natural products from TCM in modulating ferroptosis for respiratory diseases like acute lung injury (ALI), asthma, pulmonary fibrosis (PF), chronic obstructive pulmonary disease (COPD), lung ischemia–reperfusion injury (LIRI), pulmonary hypertension (PH), and lung cancer, aiming to provide new insights for research and clinical application in TCM for respiratory health.

## Background

Respiratory diseases cover a broad spectrum, from upper respiratory tract infections to serious conditions like chronic obstructive pulmonary disease (COPD), asthma, pulmonary fibrosis (PF), acute respiratory distress syndrome (ARDS), acute lung injury (ALI), lung ischemia–reperfusion injury (LIRI), pulmonary hypertension (PH), and lung cancer. These conditions, characterized by structural changes in airway and lung tissues and reduced respiratory function, pose significant health and economic burdens worldwide. Notably, COVID-19 caused 18 million deaths between 2020 and 2021, further emphasizing the global challenge of respiratory diseases [1]. COPD is the third leading cause of death globally [2], while ARDS and ALI account for no less than 4% of U.S. hospitalizations annually [3]. Lung cancer, leading in cancer-related deaths, saw 2.24 million new cases and 1.8 million fatalities in 2020, as reported by the International Agency for Research on Cancer [4]. Risk factors include smoking, air pollution, infections, and obesity [5]. Despite the availability of treatments like antibiotics and lung transplants, their side effects have prompted the search for innovative therapeutic approaches [6].

First introduced by Dixon et al. in 2012, ferroptosis is a form of programmed cell death (PCD), a term distinct from other modes of cell death such as necrosis, apoptosis, autophagy, necroptosis, and pyroptosis, which is essential for maintaining homeostatic balance [7–9]. Morphologically, ferroptosis features mitochondrial shrinkage, denser membranes, reduced mitochondrial cristae, with an intact cell membrane and normal-sized nucleus without chromatin condensation [10]. Biochemically, ferroptosis is triggered by the depletion of intracellular glutathione (GSH) and a decrease in the activity of glutathione peroxidase 4 (GPX4). This imbalance leads to lipid peroxidation, which is further exacerbated by Fe^2+^ through the Fenton reaction, generating a high concentration of reactive oxygen species (ROS) [9, 11, 12]. Ferroptosis has been implicated in a variety of multi-systemic diseases, including neurological disorders, cancers, renal trauma, and notably, pulmonary diseases [13]. Numerous studies have substantiated its role in the pathogenesis and progression of lung diseases such as lung cancer, ALI, COPD, PF, asthma, and infections [14–19]. Studies have found that the ferroptosis inhibitor ferrostatin-1 can prevent pneumonia caused by P. aeruginosa (PAO1) infection [20]. In addition, PAO1 increases the mortality of irradiated mice by inhibiting the host anti-ferroptosis system GSH/GPX4 [19]. Consequently, targeting ferroptosis presents a promising avenue for the development of innovative therapies for lung diseases.

Rooted in foundational texts like *the Huangdi Neijing* and *the Treatise on Febrile Diseases*, TCM offers preventive and therapeutic solutions using natural products with diverse pharmacological actions, including anticancer [21], anti-inflammatory [22], antioxidant [23], and immunomodulatory actions [24]. Numerous investigations have documented the extensive utilization of natural products in the treatment of diverse conditions such as malignancies, cardiovascular and cerebrovascular diseases, immune-related disorders, and neurological ailments [25–28], gaining its advantage from its capacity to act through multiple targets, pathways, and mechanisms [29].

Research has increasingly focused on TCM's protective effects against lung diseases by modulating ferroptosis, involving compounds, such as terpenes, flavonoids, phenols, polysaccharides, etc. [17, 30–33]. This review aims to summarize the research on natural products in treating lung conditions, including lung cancer, ALI, asthma, COPD, PF, LIRI, and PH, emphasizing the modulation of ferroptosis and related signaling pathways, serving as a guide for TCM application in respiratory health.

## The mechanism of ferroptosis

### Iron metabolism in ferroptosis

Iron, essential for lipid peroxide (LPO) formation and ferroptosis initiation, plays a pivotal role in oxygen transport, mitochondrial electron transfer, DNA synthesis, and other key cellular activities [34, 35]. Iron homeostasis is pivotal for various physiological functions, with Fe^2+^ ions undergoing oxidation to Fe^3+^ and then binding to transferrin (TF) in the bloodstream to form Tf-Fe^3+^ complexes. These complexes, by interacting with the membrane protein transferrin receptor 1 (TFR1), facilitate the transport of iron to different storage sites, crucial for myriad cellular activities [36–38]. Silencing the transferrin receptor (TFRC) gene, which encodes TFR1, has been shown to inhibit erastin-induced iron depletion [39]. Both TF and TFRC play essential roles in the regulation of ferroptosis by promoting the cellular uptake of iron from the extracellular milieu. Furthermore, ferritin-targeted autophagy, or ferritinophagy, leads to lysosomal degradation of ferritin, releasing intracellular iron in unstable iron pools. Excessive free iron then accelerates lipid peroxidation and the Fenton reaction, ultimately resulting in ferroptosis [40]. The degradation of ferritin is accelerated either by activating a selective cargo receptor for ferritin autophagy, nuclear receptor coactivator 4 (NCOA4), or by inhibiting the ferritin export protein, solute carrier family 40 member 1 (SLC40A1). The prostatic iron reductase six transmembrane epithelial antigen 3 (STEAP3) converts Fe^3+^ to Fe^2+^ in endosomes, facilitating iron transport to labile iron pools via divalent metal transporter 1 (DMT1) and storage in ferritin, a key cytoplasmic iron storage protein complex comprising ferritin light chain (FTL) and ferritin heavy chain 1 (FTH1) [41–43]. Under pathological conditions, ferritin releases excess Fe^2+^, which reacts with H_2_O_2_ in a Fenton reaction [44], producing hydroxyl radicals, increasing ROS, and leading to lipid peroxidation and ferroptosis initiation [45]. As a result, disruption of iron absorption, storage, utilization and efflux may lead to an imbalance in iron homeostasis, and elevated levels of Fe^2+^ lead to the generation of a substantial quantity of ROS, which disrupts intracellular redox balance, induces oxidative stress, initiates lipid peroxidation, and ultimately triggers ferroptosis [46–48].

### Lipid peroxidation

Lipid metabolism is intricately linked to the onset and progression of ferroptosis. Polyunsaturated fatty acids (PUFAs) possess bis-allylic hydrogen atoms that are readily abstracted, rendering them susceptible to lipid peroxidation. Key enzymes involved in lipid metabolism, such as arachidonic acid lipoxygenase 15 (ALOX15), acyl-CoA Synthetase long chain member 4 (ACSL4), and lysophosphatidylcholine acyltransferase 3 (LPCAT3), are requisite for the ferroptotic process. Initially, acyl-arachidonic acid (AA) and adrenaline (AdA) are activated by ACSL4 to form acyl-CoA derivatives. Subsequently, LPCAT3 esterifies these derivatives to phosphatidyl ethanolamine (PE), generating compounds like AA-PE and AdA-PE, which are finally oxidized to LPO by ALOX15 [8, 49]. Down-regulating the expression of ACSL4 and LPCAT3 genes in cellular systems can effectively inhibit the generation of LPO and enhance resistance to iron-induced cell death. Malondialdehyde (MDA) and 4-hydroxynonenal (4-HNE) are produced during the degradation of LPO, which can be detrimental to the structure and function of proteins and nucleic acids, making it essential to reduce lipid peroxidation [50, 51]. In the presence of GPX4, toxic lipid hydroperoxides (L-OOH) were converted to non-toxic lipid alcohols (L-OH), which prevented Fe^2+^-dependent accumulation of lipid ROS on membrane lipids and inhibited the production of ferroptosis [52]. These fatty acids play a crucial role in the execution of ferroptosis; therefore, the quantity and distribution of PUFAs within cells are key determinants in the extent of lipid peroxidation and, consequently, the cell's susceptibility to ferroptosis [53].

### Imbalance of the antioxidant system

GSH is a tripeptide composed of the amino acids glutamic acid (Glu), cysteine (Cys), and glycine (Gly), and serves as a crucial intracellular antioxidant [54]. A decline in GSH synthesis disrupts the intracellular redox balance, leading to the accumulation of peroxidized PUFAs. This inability to efficiently eliminate lipid peroxidation subsequently triggers ferroptosis. GSH synthesis is dependent on the cystine/glutamate antiporter system (system Xc −), a membrane-bound amino acid anti-transporter comprised of solute carrier family 7 member 11 (SLC7A11) and solute carrier family 3 member 2 (SLC3A2) [55]. SLC7A11 functions as a cystine-glutamate anti-transporter. Under pathological conditions, inhibition of the system Xc − restricts the transport of cystine into the cell, thereby reducing cysteine synthesis and consequently diminishing GSH production. This leads to the depletion of GPX4, the generation of lipid ROS, and ultimately the onset of ferroptosis [56, 57]. GPX4, or phospholipid hydroperoxide glutathione peroxidase (PHGPX), is crucial in the glutathione (GSH) antioxidant system, converting GSH to glutathione disulfide (GSSG) and turning LPO into harmless lipid alcohols to prevent lipid peroxidation from ROS [8, 57]. Lower GPX4 levels increase ferroptosis risk, while higher levels protect against it [11]. Additionally, when cysteine is scarce, gamma-cysteine ligase's catalytic unit (GCLC) activates a GSH-independent defense by utilizing an alternative amino acid to prevent ferroptosis, highlighting a non-traditional pathway for maintaining antioxidant system equilibrium [58].

### Other ways

Voltage-dependent anion channels (VDACs) are crucial for ion and metabolite transport across membranes and play a significant role in ferroptosis [59]. Erastin, a ferroptosis inducer, targets VDACs, causing mitochondrial dysfunction and a surge in ROS from mitochondria, leading to iron-dependent cell death [12]. Moreover, calcium overload can activate VDACs, increasing mitochondrial ROS and decreasing mitochondrial membrane potential (MMP). This triggers the expansion of the mitochondrial permeability transition pore (MPTP), further contributing to mitochondrial dysfunction and ferroptosis [60, 61].

Nuclear factor erythroid 2-related factor 2 (Nrf2) is a critical regulator of cellular oxidative stress and controls the expression of various antioxidant genes, including heme oxygenase-1 (HO-1), nicotinamide adenine dinucleotide phosphate (NADPH), and quinone oxidoreductase 1 (NQO1) [62]. Exposure to oxidative stress leads to increased nuclear accumulation and constitutive activation of Nrf2, which not only promotes tumor growth but also significantly contributes to treatment resistance in tumors [8]. Additionally, there is evidence that Nrf2 protects cells from ferroptosis through various pathways by regulating target genes like SLC7A11, GPX4, GSH, and ferritin [63, 64].

The tumor suppressor gene p53 indirectly influences ferroptosis by down-regulating SLC7A11, promoting its nuclear translocation [65]. Research indicates p53 pathways affect GPX4, GSH, and ROS levels, essential for ferroptosis [66]. Additionally, p53 targets the spermine/spermidine N1-acetyltransferase 1 (SAT1) enzyme, i.e., a catabolic rate-limiting enzyme, upregulating ALOX15, leading to lipid peroxidation and ferroptosis [67]. DMT1, functioning as a proton-coupled iron pump that transports iron to unstable iron pools through cell membrane potential differences, is upregulated by p53 to enhance ROS and induce ferroptosis in NSCLC [68].

## Natural products for the treatment of respiratory diseases targeting ferroptosis

### Lung cancer

**Fig. 1:**
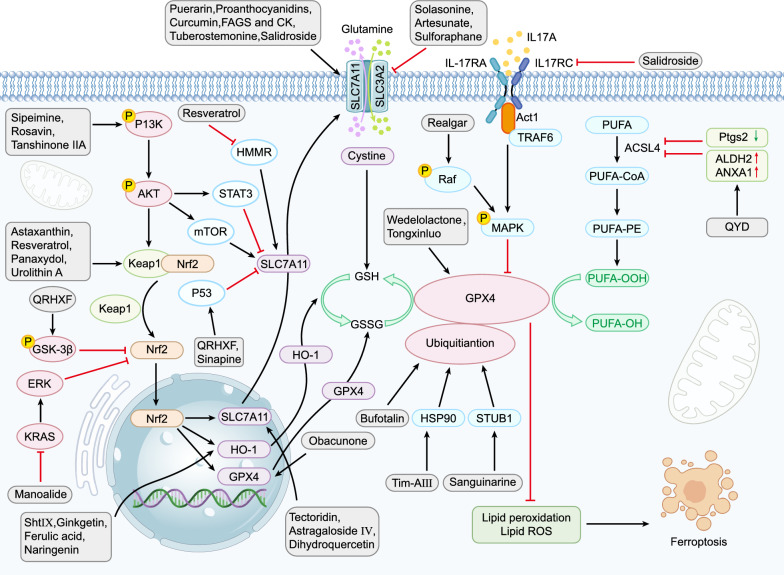
The role of SLC7A11-GSH-GPX4 axis in natural products—modulated ferroptosis in respiratory diseases. The modulations of ferroptosis by natural products in respiratory diseases are orchestrated through various mechanisms, prominently via GPX4-related pathways. These pathways crucially influence lipid peroxidation, an essential process in ferroptosis. Natural products up-regulate Nrf2 gene expression, stimulating its downstream target HO-1 and enhancing SLC7A11 protein expression. Consequently, GPX4 is activated either directly or indirectly, inhibiting ferroptosis. Moreover, multiple targets are involved in regulating the SLC7A11/GPX4 axis, including the activation of system Xc^−^ , which facilitates GSH synthesis and GPX4 activation to modulate ferroptosis. On the contrary, ACSL4 overexpression catalyzes the oxidation of PUFAs into lipid hydroperoxides. These hydroperoxides are then converted into non-toxic lipid alcohols through GPX4 activation. In the context of the immune response, Interleukin IL-17 hinders GPX4, leading to induced ferroptosis. Notations: Black Arrow (↓): Indicates promotion. Red Rough Arrow (⟂): Indicates inhibition. Green Arrow: Indicates a decrease. Red Arrow: Indicates a increase.

**Fig. 2:**
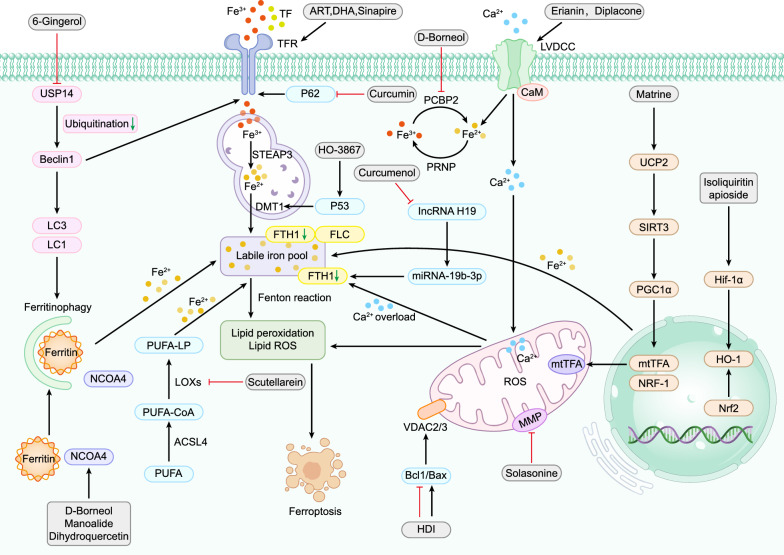
The role of iron metabolism in natural products—modulated ferroptosis in respiratory diseases. Iron metabolism is intimately linked with the mechanisms through which TCM modulates ferroptosis in respiratory diseases. An accumulation of a significant amounts of ferrous ions initiates the Fenton reaction, thereby enhancing lipid peroxidation, a pivotal step in inducing ferroptosis. Free iron binds with ferritin and is subsequently transported to the endosome through the transferrin receptor. Within the endosome, STEAP3 catalyzes the conversion of ferric iron into ferrous iron, which is then channeled into the labile iron pool via DMT1. The oxidation of PUFA coincides with the formation of ferrous ions. The influx of calcium ions causes mitochondrial calcium overload, leading to a substantial accumulation of ROS, the destruction of FLC, and FTH. These events culminate in the release of ferrous ions from the labile iron pools and the Fenton reaction, precipitating ferroptosis. Moreover, factors such as Nrf2, Hif-1α, HO-1, and mtTFA accentuate the increase of labile iron, while ferritophagy emerges as another pathway inducing ferroptosis. Notations: Black Arrows (↓): Indicate facilitation. Red Rough Arrows (⟂): Indicate inhibition. Green Arrows: Indicate a decrease

Numerous studies have highlighted the role of ferroptosis in both the etiology and treatment of various forms of cancer, including but not limited to aggressive types such as breast cancer, liver cancer, stomach cancer, rectal cancer, glioma, and pancreatic cancer [69]. Targeting ferroptosis in the context of lung cancer has the potential to mitigate disease progression and metastasis, as well as to overcome, to some extent, the drug and radiation resistance commonly exhibited by lung cancer cells. Non-small cell lung cancer (NSCLC) constitutes the predominant subtype of lung cancer, accounting for approximately 85% of cases and encompassing squamous cell carcinoma, large cell carcinoma, and adenocarcinoma [70, 71]. Consequently, contemporary research on ferroptosis in lung cancer is primarily focused on NSCLC. Chemotherapy remains the principal treatment modality in the clinical management of NSCLC, with cisplatin being the most frequently employed chemotherapeutic agent [72]. However, the emergence of cisplatin resistance poses a significant challenge to achieving optimal therapeutic outcomes in patients undergoing chemotherapy for lung cancer. Natural products have gained prominence as a valuable adjunct in the comprehensive treatment of various malignancies. Besides, natural products have been shown to positively impact the quality of life and extend the survival duration of patients with advanced lung cancer, irrespective of whether conventional treatments are administered [73]. A summary of natural products used in lung cancer therapy, their primary sources, mechanisms of targeting ferroptosis, and main effects can be found in Table 1. Additionally, we analyzed and summarized the targets and signaling pathways of natural products targeting ferroptosis in the treatment of lung diseases, as shown in Figs. 1, 2.

**Table 1:** Natural products targeting ferroptosis in lung cancer

Component	Classification	Main roots	Test models	Dose	Mechanisms	Specific effects	Refs.
Solasonine	Alkaloids	*Solanum* *nigrum* L.	Calu-1 and A549 cells	In vitro: 10, 15, 20 μM (calu-1); 20, 25, 30 μM (A549)	Causing GSH redox system imbalance and mitochondrial oxidative stress	Causing iron overload and redox imbalance; lipid peroxidation; mitochondrial damage; the destruction of the GSH redox system: decreasing expression of GPX4, SLC7A11, GSH, and Cys; MMP hyperpolarization	[74]
Erianin	Phenols	Dendrobium	H460 and H1299 cells; Balb/c nude mice	In vitro: 12.5, 25, 50, 100 nm; In vivo: 100 mg/kg	Inducing Ca^2+^/ CaM signal pathway	Promoting cell cycle arrest in G2/M; activating CAM and regulating L-type voltage-dependent Ca^2+^ channels; lipid peroxidation; promoting the production of ROS, MDA, TRF; decreasing expression of GPX4, CHAC2, SLC40A1, SLC7A11, HO-1, GSH	[75]
Diplacone	Flavonoids	Paulownia tomentosa mature fruit	A549 cells	In vitro: 40 μM	Increasing mitochondrial Ca^2+^ Influx and MPTP	Increasing the level of intracellular Ca^2+^, mitochondrial ROS, and mitochondrial Ca^2+^ overload; increasing the opening of the VDAC and MPTP; inducing loss of MMP; lipid peroxidation	[76]
Qingrehuoxue Formula	Formulas	Chinese herbal medicine	male Balb/c nude mice	In vivo: 15 g/kg	Upregulating P53 and GSK-3β and downregulating Nrf2 signal pathways	Increasing the levels of intracellular ROS, Fe^2+^, H_2_O_2,_ GSH and MDA↑; decreasing the expression of SLC7A11, GPX4; shrunking mitochondria with increasing membrane density and decreasing or disappearing mitochondrial cristae	[78]
Bufotalin	Steroids	Venenum bufonis	A549 cells; male Balb/c nude mice	In vitro: 4 μM; In vivo: 5/10 mg/kg	Facilitating the ubiquitination and degradation of GPX4	Increasing the level of lipid ROS, 4-HNE, MDA, Fe2 + ; decreasing the ratio of GSH/GSSG and NADPH/NADP +	[32]
Dihydroisotans-hinone I	Quinones	Salvia miltiorrhiza Bunge	A549, H460 and IMR-90 cells; xenograft nude mice	In vitro: 20–30 μM; In vivo: 30 mg/kg	Blocking the protein expression of GPX4	Increasing the level of lipid ROS and MDA; decreasing expression of GPX4 and GSH	[80]
Sanguinarine	Alkaloids	Sanguinaria canadensis Linn	A549 and H3122 cells; xenograft mice	In vitro: 10 μM; In vivo: 5 mg/kg	Decreasing the protein stability of GPX4 through E3 ligase STUB1-mediated ubiquitination and degradation of GPX4	Increasing Fe^2+^ concentration, ROS level, and MDA content; decreasing GSH content	[81]
Red ginseng polysaccharide	Polysaccharides	Panax ginseng	A549 and MDA-MB-231 cells	In vitro: 200 μg/ml	Blocking the protein expression of GPX4	Increasing the release of LDH and the level of lipid ROS; decreasing expression of GPX4	[82]
Timosaponin AIII	Steroids	Anemarrhena Asphodeloides Bunge	H1299, A549, SPC-A1 and LLC cells; male C57BL/6 J or Balb/c- nude mice	In vitro: 4 μM; In vivo: 12.5 mg/kg (low-dose), 50 mg/kg (high-dose)	Facilitating HSP90 mediated GPX4 ubiquitination and degradation	Suppressing cell proliferation and migration, inducing G2/M phase arrest; increasing the levels of iron, lipid ROS, MDA, HMOX-1; decreasing expression of GSH, FTL, GPX4, SLC40A1, SLC7A11; inducing loss of MMP	[83]
Zerumbone	Terpenoids	Zingiber zerumbet rhizomes	HPAEpiC, A549, and H460 cell; BALB/c nude mice	In vitro: 100 μM; In vivo: 20 mg/kg (low-dose), 40 mg/kg (high-dose)	Downregulating AKT/STAT3/SLC7A11 axis	Increasing the level of MDA; decreasing the levels of GSH, GPX4 and SLC7A11	[86]
S-3′-hydroxy-7′, 2′, 4′-Trimethoxyisoxane	Flavonoids	Dalbergia odorifera T. Chen	A549 and H460 cells; Balb/c nude mice	In vitro: 16 μM; In vivo: –	Inhibiting Nrf2/HO-1 signaling pathway	Increasing the level of Fe2 + , ROS and MDA; decreasing the levels of GSH, GPX4, p21, FTH1, Nrf2, HO-1; TEM: cell membrane rupture, mitochondrial shrinkage, thickening of the mitochondrial membrane density, and diminished or disappeared mitochondrial ridges	[88]
Ginkgetin	Flavonoids	Ginkgo biloba leaves	Xenograft nude mice	In vitro: 5 μM; In vivo: 30 mg/kg	Inhibiting Nrf2/HO-1 signaling pathway	Increasing labile iron pool and lipid peroxidation; decreasing expression of SLC7A11, GPX4, GSH; inducing loss of MMP	[89]
Manoalide	Terpenoids	Sponges	A549, H157, HCC827, and PC9 cells	In vitro: 15 μM	Suppressing the KRAS-ERK pathway and the Nrf2-SLC7A11 axis, mitochondrial Ca2 + overload induced-FTH1 pathways	Inducing ER stress; promoting the accumulation of lipid droplets, ROS, lipid peroxidation, mitochondria Ca2 + and iron; increasing the oxygen consumption rate and inhibiting mitochondria fatty acid oxidation; decreasing expression of Nrf2, SLC7A11, FTH1, GPX4, KRAS, P-ERK/ERK; increasing expression of NCOA4 and P-AMPK/AMPK	[90]
Hedyotisdiffusa injection	Other	Chinese herbal medicine	A549 and H1975 cells; Balb/c nude mice xenograft model	In vitro: 30 μM (A549), 40 μM (H1975); In vivo: 15 mg/kg	Regulating Bax/Bcl2/VDAC2/3 axis	Regulating VDAC2/3 activity by promoting Bax via inhibiting Bcl2; increasing the expression of 4-HNE, TFR, and HMOX1	[91]
D-Borneol	Terpenoids	Cinnamomum cam phora (L.) J. Presl	H460/CDDP cells; Xenograft tumor mice	In vitro: 2 μg/ml; In vivo: 30 mg/kg (low-dose), 60 mg/kg (high-dose)	Promoting NCOA4-mediated ferritinophagy	Increasing the level of ROS, MDA; decreasing expression of GSH, SOD, Trx, HO-1	[92]
Artesunate	Terpenoids	Artemisinin	NCI-H1299, A549, LTEP-a-2, NCI-H23, and NCI-H358 cells	In vitro: 10/30 μM	Inhibiting system Xc − and activating TFRC	Increasing the ROS level and the mRNA level of TFRC; decreasing the protein level of VDAC and SLC7A11;	[93]
Dihydroartemisinin	Terpenoids	Artemisinin	NCI-H1299, A549, LTEP-a-2, NCI-H23, and NCI-H358 cells	In vitro: 10/30 μM	Inhibiting system xc − and activating TFRC	Increasing the ROS level and the mRNA level of TFRC; decreasing the protein level of VDAC and SLC7A11;	[93]
Curcumenol	Terpenoids	Wenyujin	CCD19, H1299, H460, BEAS-2B and 293 T cells	In vitro: 300 μg/ml; In vivo: 200 mg/kg	Suppressing lncRNA H19/miR-19b-3p/FTH1 axis	Increasing the level of iron, lipid ROS, HO-1, MDA, TF; decreasing the level of GSH, Nrf2, GPX4, SLC7A11, SLC40A1, FTH1	[96]
Sulforaphane	Glycosides	Cruciferous vegetables	NCI-H69, NCI-H82 and NCI-H69AR cells	In vitro: 20 μM	Inhibiting system Xc −	Decreasing the level of SLC7A11, GSH; increasing the level of Fe2 + , lipid peroxidation	[97]
Sinapine	Alkaloids	Rapeseed and cruciferous plant species	A549, SK, H66, H460 and HBE cells	In vitro: 20 μM	Upregulating p-53,TF, TFRC; downregulating SLC7A11	Increasing intracellular ferrous iron, lipid peroxidation, MDA and ROS; decreasing the expression of SLC7A11, GSH, GPX4	[101]
HO-3867	Other	Curcumin analogs	H460, PC-9, H1975, A549, H1299, A549 p53 KO cells and H460 p53 KO cells	In vitro: 40 μM	Activating the p53-DMT1 axis and suppressing GPX4	Increasing the level of iron, ROS; increasing expression of P53, DMT1; decreasing expression of SLC7A11, GPX4	[102]
6-Gingerol	Phenols	Ginger	A549 and CCD19-Lu cells; Balb/c nude mice	In vitro: 20, 40, 80 μM; In vivo: 0.25 mg/kg (low-dose), 0.5 mg/kg (high-dose)	Inhibiting USP14-mediated Beclin1 ubiquitination, enhancing autophagy-dependent ferroptosis	Increasing level of MDA, iron and TfR1; decreasing level of USP14, FTH1, GPX4, ATF4, SOD; increasing autophagy related proteins level of Beclin- 1, NCOA4, LC3 I, LC3 II	[103]
Realgar	Other	Sulfide minerals	H23 cells	In vitro: 2 μg/ml	Suppressing the KRAS/Raf/MAPK pathway	Increasing the level of MDA, Fe2 + , ROS; decreasing expression of GSH; inducing loss of MMP	[106]
Curcumin	Phenols	Turmeric plant	A549 and H1299 cells; female C57BL/6 mice	In vitro: 30 μM; In vivo: 100 mg/kg	Activating autophagy-dependent ferroptosis	Increasing the level of iron, lipid peroxidation, ROS, MDA, IREB2, ACSL4; decreasing the level of SOD, GSH, SLC7A11, GPX4; inducing mitochondrial membrane rupture; decreasing mitochondrial cristae; increasing autolysosome; increasing autophagy related proteins level of Beclin1 and LC3, and decreasing the level of P62	[108]
Resveratrol	Phenols	Peanuts, grapes, knotweed, mulberries	H520 cells	In vitro: 50 μmol/L;	Regulating SLC7A11-HMMR interaction, enhancing the cytotoxic effect of CD8 + T cells	Increasing the level of MDA, ACSL4, TFRC; decreasing the level of GPX4, SLC7A11, HMMR, GSH, and SOD; promoting the release of TNF-α, IFN-γ, IL-12, and IL-2; enhancing the cytotoxic effects of CD8 + T cells	[111]
Resveratrol	Phenols	Peanuts, grapes, knotweed, mulberries	BEAS-2B cells	In vitro: 10 μM;	Activating the Nrf2/Keap1 signaling pathway	Decreasing reactive oxygen species production and iron deposition; increasing the expression of GPX4 and GSH	[112]

Solasonine (SS), a glycoalkaloid from *Solanum nigrum L,* demonstrates potential in cancer therapy, showing antitumor effects on lung cancer cells. Its action involves inducing ferroptosis, marked by increased levels of LPO, iron, and ROS. The effectiveness of SS is attributed to compromised antioxidant defenses and mitochondrial damage, crucial factors in the ferroptosis process it triggers [74]. Erianin, a phenolic natural product isolated from *Dendrobium chrysotoxum Lindl*, has been shown to inhibit the growth of H460 and H1299 cell lines through the induction of Ca^2+^/calmodulin (CaM)-dependent ferroptotic cell death. This process is accompanied by the formation of ROS, lipid peroxidation, and depletion of GSH [75]. Diplacone (DP), a flavonoid derivative, has been investigated for its capacity to augment mitochondrial calcium influx, ROS generation, the opening of the MPTP, and a reduction of MMP, which are characteristics of ferroptosis. Studies have established that the application of DP to A549 cells not only inhibits cell growth but also enhances lipid peroxidation, a critical step in ferroptosis, along with an increase in ATF3 expression. ATF3 has been identified as playing a role in ferroptosis by regulating the expression of genes involved in iron metabolism and lipid peroxidation. Furthermore, it has been demonstrated that ferroptosis inhibitors, such as ferrostatin-1 and liproxstatin-1, can mitigate DP-mediated cell death in A549 cells. Overall, these findings support the hypothesis that DP can induce ferroptosis in the treatment of NSCLC [76]. The Qingrehuoxue Formula (QRHXF), a two-herb Chinese medicinal formula consisting of Radix Paeoniae Rubra and Scutellaria baicalensis, contains various active compounds including baicalin and paeoniflorin [77, 78]. QRHXF treatment significantly elevates ROS, Fe^2+^, H_2_O_2_, and MDA levels, while reducing GSH levels, indicating its potent effect on oxidative stress. It suppresses the expression of SLC7A11 and GPX4, key ferroptosis markers, and induces changes in the mitochondrial ultrastructure of tumor cells without causing toxicity in tumor-bearing mice. Furthermore, QRHXF upregulates p53 and phospho-glycogen synthase kinase-3 (p-GSK-3β) expressions while downregulating Nrf2 levels. Thus, QRHXF hinders NSCLC cell progression by promoting iron-induced apoptosis and ferroptosis through the p53 and GSK-3β/Nrf2 signaling pathways [78].

Bufotalin, a steroid compound extracted from *Venenum Bufonis*, has demonstrated significant anticancer properties [79]. Research shows that bufotalin triggers ferroptosis in NSCLC cells through enhanced lipid peroxidation, driven by GPX4 degradation and elevated intracellular Fe^2+^ levels [32]. Dihydroisotanshinone I (DT), a quinone derivative isolated from the dried roots of *Salvia miltiorrhiza Bunge*, has shown inhibitory effects on the proliferation of A549, H460, and IMR-90 lung cancer cell lines. Mechanistically, DT inhibits the production of GPX4, thereby initiating ferroptosis via lipid peroxidation [80]. Sanguinarine (SAG), a benzophenanthridine alkaloid derived from the root of *Sanguinaria canadensis Linn*, exhibited significant inhibitory effects on the growth and metastasis of NSCLC in a xenograft model [81]. SAG destabilizes GPX4 through E3 ligase STUB1-mediated ubiquitination, leading to GPX4 degradation and subsequent ferroptosis [81]. Following this, Red Ginseng Polysaccharide (RGP), polysacchride, an active component of Panax ginseng C. A. Meyer (Araliaceae), has been shown to inhibit the proliferation of human A549 and MDA-MB-231 cells, induce lactate dehydrogenase (LDH) release, promote ferroptosis, and suppress GPX4 expression [82]. Similarly, Timosaponin AIII (Tim-AIII), a steroidal saponin from *Anemarrhena Asphodeloides Bunge*, induces NSCLC cell death and G2/M arrest. It achieves this therapeutic effect by interacting with its target protein HSP90, facilitating the ubiquitination and subsequent degradation of GPX4, thereby inducing ferroptosis [83]. Zerumbone, a terpenoid compound, primarily extracted from *Zingiber zerumbet Smith*, acts as an anticancer agent by inhibiting tumor proliferation and promoting cell death [84, 85]. When combined with gefitinib, Zerumbone inhibits lung cancer cell proliferation through multiple mechanisms, including the activation of the AKT/STAT3/SLC7A11 axis, which decreases GPX4 activity and thereby induces ferroptosis [86]. Nrf2 plays a critical role in maintaining cellular redox balance by activating endogenous antioxidant response elements [87]. HO-1 is the primary protein targeted by Nrf2 in the context of oxidative stress. Recent studies have emphasized the importance of Nrf2 and HO-1 in the ferroptotic response. For instance, S-3'-hydroxy-7', 2', 4'-trimethoxyisoxane (ShtIX), a novel flavonoid compound, has been shown to initiate ferroptosis in NSCLC cells by inhibiting the Nrf2/HO-1 signaling pathway [88]. Ginkgetin has been reported to induce ferroptosis in NSCLC by inactivating the Nrf2/HO-1 signaling pathway, thereby enhancing the therapeutic efficacy of cisplatin (DDP) [89]. Additionally, Sanguinarine amplifies MMP loss and DDP-induced apoptosis in NSCLC cells, supporting the potential for combining natural products with chemotherapeutic agents for tumor treatment [72]. Manoalide (MA), a marine terpenoid derived from sponges, has been observed to inhibit the proliferation of KRAS-mutated lung cancer cells and organoids. Notably, MA induces ferroptosis by inhibiting the Nrf2-SLC7A11 axis and ferritin heavy chain 1 (FTH1) pathways, which are activated by excess mitochondrial Ca^2+^. This enhances the susceptibility of osimertinib-resistant lung cancer cells to osimertinib [90].

In vitro studies have demonstrated that Hedyotis diffusa injection (HDI) can reduce the viability of lung adenocarcinoma cells and induce ferroptosis by modulating VDAC2/3 activity, which is achieved through the upregulation of pro-apoptotic protein Bax and the downregulation of anti-apoptotic protein Bcl2 [91]. Natural borneol (d-borneol), another terpenoid, is extracted from the fresh leaves and branches of *Cinnamomum camphora (L.) J. Presl*. When combined with cisplatin, d-borneol has been shown to reduce both the volume and weight of tumors, thereby exhibiting anticancer effects. Mechanistically, its role has been linked to ferroptosis, NCOA4-mediated ferritin autophagy, and the upregulation of prion protein (PRNP). Additionally, it leads to the downregulation of Poly(rC)-binding protein 2 (PCBP2), resulting in elevated intracellular iron ion levels [92].

The anti-cancer properties of artemisinin derivatives, such as artesunate (ART) and dihydroartemisinin (DHA), have gained considerable attention in the medical field for their efficacy against various cancers, including lung cancer, colon cancer, nasopharyngeal cancer, and glioma. Both ART and DHA are terpenoid derivatives of artemisinin and have been shown to downregulate the expression of the cystine/glutamate transporter, a critical inhibitor of ferroptosis in NSCLC cells. These compounds primarily induce ferroptosis by upregulating the expression of TFRC, a marker indicative of ferroptosis [93]. Non-coding RNAs, particularly long non-coding RNAs and microRNAs, are implicated in various biological processes, including apoptosis, autophagy, and tumor initiation [94]. FTH1 serves as a marker for ferroptosis. Curcumenol, a terpenoid compound found in *Wenyujin*, has demonstrated significant anti-cancer properties across various cancer types [95]. Studies have shown that curcumenol-induced ferroptosis is the primary mechanism of lung cancer cell death, both in vitro and in vivo. The lncRNA H19/miR-19b-3p/FTH1 axis plays a crucial role in this ferroptotic cell death induced by curcumenol [96]. Sulforaphane (SFN), a glycoside derived from cruciferous vegetables, has been shown to decrease the expression of SLC7A11, a key component of the system Xc^−^. This reduction suggests that the anti-tumor effects of SFN may be attributed to the induction of ferroptosis in SCLC cells, potentially due to the downregulation of SLC7A11 at both mRNA and protein levels [97].

Sinapine (SI) is an alkaloid extractable from various rapeseed and cruciferous plant species [98]. Numerous studies have attested to its antioxidant, neuroprotective, anti-inflammatory, and anti-tumor properties [99, 100]. The p53 protein functions as a transcription factor that inhibits cell proliferation and viability, acting as a pivotal tumor suppressor and a ferroptosis regulator [73]. Researchers have confirmed that SI induces ferroptosis in NSCLC cells through a mechanism that involves p53-dependent downregulation of SLC7A11 and upregulation of TF and TFR, ultimately leading to iron accumulation and ferroptosis [101]. HO-3867, a synthetic analog of curcumin (CUR), exhibits potent antitumor activity against various cancer cell types. This compound induces ferroptosis via the activation of the p53-mediated signaling pathway, targeting DMT1 as its downstream effector and concurrently inhibiting the expression of GPX4 [102].

6-Gingerol, a naturally occurring phenol found in ginger, exhibits anti-tumor properties by targeting ubiquitin-specific protease 14 (USP14), a cysteine protease involved in deubiquitination that suppresses autophagy in various cancers. By downregulating USP14, 6-Gingerol enhances autophagosome formation, increases ROS and iron levels, thereby reducing survival, proliferation, and tumor size [103].

KRAS, a key lung tumor growth biomarker, presents a viable target for NSCLC therapies [104]. Activation of the Ras/Raf/ERK pathway is essential for cancer progression. In the caenorhabditis elegans model, realgar, a sulfide mineral from ores, downregulates Ras expression through the Ras/MAPK signaling pathway [105]. Further studies reveal Realgar's potential to inhibit KRAS-mutated lung cancer cell growth by inducing ferroptosis via the Raf-mediated Ras/MAPK pathway [106], positioning it as a promising anti-cancer agent, especially for Ras mutation-targeted ferroptosis.

Curcumin, a phenolic compound from turmeric, is recognized for promoting ferroptosis, particularly in NSCLC, by activating autophagy. This mechanism, linked to the maintenance of cellular iron homeostasis by ferritin [107], suggests that inducing ferroptosis through autophagy can improve NSCLC treatment outcomes [108]. The interplay between autophagy and ferroptosis highlights the potential of leveraging natural products for developing multi-pathway disease treatments.

Anti-cancer immune responses: in-depth exploration have led to the classification of NSCLC, specifically lung squamous carcinoma (LUSC), as an "immunotherapy-responsive disease" [109]. Mutations affecting cellular iron levels within tumor cells have the potential to trigger robust anti-tumor immune responses both in vivo and in vitro, thereby potentially enhancing the efficacy of immune checkpoint inhibitors [110]. Resveratrol, a phenolic compound, concentrated in the peanuts, grapes, knotweed, mulberries, has been shown to induce higher levels of ferroptosis in H520 cells, improve the cytotoxic effects of CD8^+^ T cells within the tumor microenvironment by modulating the HMMR/ferroptosis axis in cases of LUSC [111]. However, in erastin-induced ferroptosis in BEAS-2B cells, resveratrol promotes GPX4 and GSH expression and protects BEAS-2B cells from ferroptosis via the Nrf2/Keap1 pathway [112].

To summarize, the reviewed studies demonstrate the efficacy of 8 natural products from herbs—flavonoids, phenols, alkaloids, terpenoids, steroids, quinones, polysaccharides, and glycosides—comprising 21 active ingredients. These compounds modulate ferroptosis, inhibit tumor growth, invasion, metastasis, and enhance cancer survival. They induce ferroptosis through mechanisms like increased GPX4 ubiquitination, GPX4 and GSH depletion, calcium channel activation leading to calcium overload, iron metabolism enhancement, ferritin autophagy initiation, Fenton reaction, mitochondrial membrane disruption, ROS release, and lipid peroxidation. Key pathways include GPX4-related, SLC7A11-related, VDAC-mediated, p53-mediated, Nrf2-mediated, and NCOA4-mediated mechanisms. But it should be noted that balancing the effects of ferroptosis-modulating drugs on cancerous versus healthy tissues remains a significant challenge.

### Acute lung injury

ALI is a critical condition that may manifest as a severe form of ARDS or part of Multiple Organ Dysfunction Syndrome (MODS). It’s typically marked by uncontrolled oxidative stress, pulmonary inflammation, damage to the alveolar and microvascular endothelia, and pulmonary edema [113], with the potential to evolve into ARDS and MODS. Current treatment modalities for ALI primarily include nutritional support, mechanical ventilation, etiological treatment, symptomatic relief, and maintenance of internal homeostasis, supplemented with glucocorticoid hormone, inhaled pulmonary vasodilator, nerve muscle blocker [114, 115]. Given the high morbidity and mortality associated with ALI, there's a pressing need for new therapeutic approaches. Recent research highlights that bioactive compounds from Chinese herbs and their extracts could offer new pathways to mitigate ALI/ARDS. Notably, increased iron accumulation has been observed in the lungs of mice suffering from ALI. Excessive iron promotes the generation of superoxide and induces lipid peroxidation through the Fenton reaction, ultimately triggering ferroptosis [66]. Ferroptosis has been implicated in several models of ALI, including those induced by lipopolysaccharide (LPS), intestinal ischemia/reperfusion (I/R), seawater drowning, fine particulate matter (PM2.5), oleic acid, and Pseudomonas aeruginosa (PA) [13]. We collected relevant lung injury studies and found that inhibition of ferroptosis has a significant effect on the treatment of ALI.

Nrf2, a key transcription factor, is essential in regulating cellular antioxidant defenses and plays a vital role in mitigating ALI by preventing ferroptosis. Its activation leads to a decrease in GSH depletion and an increase in the expression of oxidative stress-related factors, including hypoxia-inducible factor 1α (HIF-1α) and HO-1. This activation subsequently inhibits the accumulation of MDA, ROS, and lipid ROS, enhances mitochondrial structure and function, reduces ferroptosis, and alleviates ALI [89, 116]. HIF-1α plays a crucial role in bolstering anti-ferroptotic defenses, reducing iron accumulation, and boosting GPX4 expression [117]. The Nrf2/HO-1 signaling pathway is pivotal in controlling cellular damage caused by various factors, with its activation offering protection against tissue and cellular damage through diverse mechanisms [118]. SLC7A11, also known as xCT, alleviates oxidative stress in epithelial cells by enhancing intracellular cystine levels, acting as a negative feedback loop to restrain the Nrf2/HO-1 pathway, thus preserving cellular antioxidant balance [119]. Collectively, these studies unequivocally establish that Nrf2 serves as a major negative regulator of ferroptosis in ALI, and that ferroptosis itself contributes to the progression of ALI. In this review, we summarize the mechanisms by which natural products treat ALI through the regulation of ferroptosis, as detailed in Table 2.

**Table 2:** Natural products targeting ferroptosis in ALI

Component	Classification	Main roots	Test models	Dose	Mechanisms	Specific effects	Refs.
Astaxanthin	Terpenoids	Various microorganisms, phytoplankton, marine animals, and seafood	In vitro: LPS induced RAW264.7 cells; In vivo: LPS induced female Balb/c mice	In vitro: 5, 10, 20 μM; In vivo: 20 mg/kg	Activating the Keap1-Nrf2/HO-1 pathway	Decreasing inflammatory relative: COX2, iNOS, NO↓; NF-KB, P-P65↓; decreasing lipid metabolism relative: lipid ROS↓; inhibiting ferroptosis relative: 4-HNE, PTGS2, ACSL4 and CD68↓; SLC7A11, GPX4 and FTH1↑	[121]
Panaxydol	Polyacetylenes	Panax ginseng	In vitro: LPS induced BEAS-2B cells; In vivo: LPS induced male C57BL/6 mice	In vitro: 40 μg/ml; In vivo: 20 mg/kg	Activating the Keap1-Nrf2/HO-1 pathway	Decreasing inflammatory relative: TNF-α, IL-1β, and IL-6↓; MPO activity, neutrophil percentage (%) ↓; reducing pulmonary edema: Lung W/D ratio, total protein↓; inhibiting ferroptosis relative: Fe2 + , MDA ↓; GSH and GPX4 ↑	[30]
Urolithin A	Phenols	A secondary metabolite of ellagitannins and ellagic acid	In vitro: LPS induced BEAS-2B cells; In vivo: LPS induced male C57BL/6 mice	In vitro: 10 μM; In vivo: 50 mg/kg	Activating the Keap1-Nrf2/HO-1 pathway	Decreasing inflammatory relative: TNF-α, IL-1β, and IL-6↓; neutrophil percentage (%) ↓; reducing pulmonary edema; Lung W/D ratio, total protein↓; reducing oxidative stress: Intracellular ROS and mitochondrial ROS, MDA↓; GSH, CAT, SOD↑; inhibiting ferroptosis relative: GPX4, SLC7A11↑; Fe2 + , 4-HNE↓; the number of mitochondria↑, mitochondria structural damage↓	[128]
Obacunone	Flavonoids	Citrus and rutaceae species	In vitro: LPS induced BEAS-2B cells; In vivo: LPS induced male C57BL/6 mice	In vitro: 20 μM; In vivo: 2.5, 5, 10 mg/kg	Activating the Nrf2/SLC7A11/GPX4 axis	Decreasing inflammatory relative: IL-1β, IL-6, TNF-α↓; KL-6, CRP and neutrophils (%) ↓; lymphocytes (%) ↑; reduced the LPS-induced loss of ALI lung tissue structure loss, apoptosis injury, and edema; reducing oxidative stress: CAT, GSH, SOD↑; MDA↓; inhibiting ferroptosis relative: Fe 2 + , 4-HNE↓; GPX4, SLC7A11↑; TEM: mitochondrial structural damage	[131]
Wedelolactone	Lactones	Eclipta prostrata	In vitro: LPS induced AR42J cells; In vivo: sodium taurocholate or caerulein induced male Sprague–Dawley rats	In vitro: 20 μM; In vivo: 20, 50 mg/kg (taurocholate-induced), 50, 100 mg/kg (caerulein-induced)	Activating GPX4 level	Decreasing proinflammatory cytokines: TNF-α, IL-1β, IL-18, NLRP3↓; reducing oxidative stress: ROS, MDA↓; inhibiting lipid peroxidation and ferroptosis: GSH, GSH-Px, GPX4, GSDMD, DGSDMD-N↑, 4-HNE↓; decreasing serum pancreatic digestive enzymes: LDH, amylase, lipase↓; inhibiting pyroptosis: caspase1, caspase11↓	[133]
Qingyi Decoction	Formulas	Chinese herbal medicine	In vivo: Sodium taurocholate induced Aprague-Dawley male rats	In vivo: 10 g/kg	Activating ALDH2/ANXA1; downregulating ICAM-1	Decreasing inflammatory relative: TNF-α and IL-6↓; inhibiting the increase of serum amylase and Lung W/D ratio; reducing neutrophil infiltration: ANXA1↑, ICAM-1, P-P65/P65↓; inhibiting ferroptosis relative: Fe2 + , MDA, MPO↓; ALDH2, GSH, SLC7A11, FTH1 and GPX4↑	[134]
Matrine	Alkaloids	Sophora flavescens	In vitro: LPS-induced BEAS-2B cells and MLE-12 cells; In vivo: cerulein and LPS induced UCP2 -/- mice	In vitro: -; In vivo: 200 mg/kg	Activating the UCP2/SIRT3/PGC1αpathway	Decreasing inflammatory cytokines: IL-6, IL-1β, and TNF-α, total BALF protein↓; reducing lipid peroxidation: intracellular ROS, MPO↓; inhibiting ferroptosis: Fe2 + , MDA, ACSL4↓; GSH, GPX4, NRF1, mtTFA, HO-1 and NQO1↑	[136]
Sipeimine	Alkaloids	Fritillaria roylei	In vivo: PM2.5 dust suspension induced male Sprague–Dawley rats	In vivo: 15 mg/kg (low-dose), 30 mg/kg (high-dose)	Activating the PI3K/Akt/Nrf2 pathway	Decreasing inflammatory cytokines: TNF-α and IL-1β↓; inhibiting ferroptosis relative: MDA, 4-HNE, iron↓; Nrf2, GSH, GPX4, HO-1, SLC7A11 and FTH1↑; the mitochondria ultrastructure was significantly improved	[140]
Tectoridin	Flavonoids	The rhizome of Belamcanda chinensis	In vitro: PM2.5-induced BEAS-2B cell; In vivo: PM2.5-induced Nrf2-knockout mice	In vitro: 100 μM; In vivo: 50 mg/kg (low-dose), 100 mg/kg (high-dose)	Activating the Nrf2/SLC7A11/GPX4 axis	Decreasing inflammatory factors, lipid peroxidation, iron accumulation and ferroptosis: MDA↓, GSH, GPX4, xCT, FTH1/FTL, TFR↑	[141]
Rosavin	Glycosides	Rhodiola plants	In vivo: PM2.5 dust suspension induced male Sprague–Dawley rats	In vivo: 50 mg/kg (low-dose), 100 mg/kg (high-dose)	Activating the PI3K/Akt/Nrf2 pathway	inhibiting ferroptosis relative: MDA, 4-HNE, iron↓; Nrf2, GSH, GPX4↑	[142]
Astragaloside IV	Glycosides	Astragalus	In vivo: PM2.5 dust suspension induced C57BL/6 J male mice	In vivo: 50 mg/kg (low-dose), 100 mg/kg (high-dose)	Activating the Nrf2/SLC7A11/GPX4 axis	Reducing pulmonary edema; reducing oxidative stress: MDA and MPO↓; SOD↑; decreasing inflammatory cytokines: IL-6, TNF-α, IL-1β and COX2↓; inhibiting ferroptosis relative: Nrf2, HO-1, SLC7A11, GPX4, FLC, FTH1↑; TFRC↓; the mitochondria ultrastructure was significantly improved	[143]
Isoliquiritin apioside	Flavonoids	Glycyrrhizae radix et rhizoma	In vitro: Hypoxia and reoxygenation induced MLE-2 cells; In vivo: I/R induced male C57BL/6 mice	In vitro: 25, 50, 100 μM; In vivo: 50 mg/kg (low-dose), 100 mg/kg (high-dose)	Inhibiting Hif-1α/HO-1 pathway	Decreasing proinflammatory cytokines: TNF-α, IL-6, Hmgb1↓; inhibiting ferroptosis: MDA, Fe2 + , Ptgs2, ACSL4↓; GSH, GPX4↑	[148]
Salidroside	Glycosides	Rhodiola rosea	In vivo: Hyperoxia-induced KM mice	In vivo: 100 mg/kg	Inhibiting the Act1/TRAF6/p38 MAPK pathway	Decreasing inflammatory and immunity relative: IL-6, TGF-β, IL-17A, IL-17RA↓; inhibiting ferroptosis relative: Fe 2 + , MDA↓; GPX4↑; reducing pulmonary edema, atelectasis, necrosis, alveolar and interstitial inflammation, and collagen deposits	[151]
Ferulic acid	Phenols	In various kinds of plants and vegetables such as tomatoes, sweet corn and rice bran	In vitro: LPS induced MLE-12 cells; In vivo: female Balb/c mice were induced by the CLP	In vitro: 0.1 μM; In vivo: 100 mg/kg	Activating the Nrf2/HO-1 pathway	Ameliorating barrier dysfunction and pulmonary edema: Lung W/D ratio, total protein↓; ZO-1, occludin, and claudin-1, TEER↑; FITC-dextran flux↓; inhibiting ferroptosis relative: ROS, MPO, Fe2 + , MDA↓; GSH, GPX4↑	[156]
Puerarin	Flavonoids	Gegen	In vitro: LPS induced A549 cells	In vitro: 80 μM	Activating SLC7A11/ GPX4 axis and FTH1	Decreasing inflammatory relative: TNF-α, IL-8, and IL-1β↓; decreasing lipid peroxidation: MDA, ROS↓; inhibiting ferroptosis relative: total iron levels and ferrous iron, NOX1↓; SLC7A11, GPX4, GSH, FTH1↑	[157]
Tripterygium wilfordii Hook.f	Terpenoids	Celastraceae plants	In vivo: Male Balb/c mice were induced by PQ	In vivo: 10 g/kg	Modulating the Keap1/Nrf2/HO-1 pathway	Reducing the levels of proinflammatory cytokines: IL-6 and TNF-α; alleviating oxidative stress: MDA↓; GSH, SOD↑	[161]
Proanthocyanidins	Flavonoids	Carthamus tinctorius L	In vivo: Mice were infected by IAV and HINI	In vivo: 20 mg/kg	Inhibiting the TGF-β1/Smad signaling pathway and IFN-γ expression	Decreasing the levels of MDA and ACSL4; upregulating the expression of GSH, GPX4, and SLC7A11;	[162]
Naringenin	Flavonoids	Citrus fruits	In vitro: AgNPs induced BEAS-2B cells; In vivo: AgNPs suspension induced male ICR mice	In vitro: 25, 50, 100 μM; In vivo: 25, 50, 100 mg/kg	Activating the Nrf2/HO-1 pathway	anti-inflammation, anti-oxidative stress, anti-apoptosis: BAX, CytC, Caspase9, Caspase3↓; Bcl2↑; anti-ferroptosis; decreasing the levels of white blood cells, neutrophils, and lymphocytes in the blood, ameliorating lung injury, suppressing the release of pro-inflammatory cytokines;	[164]

↑: up-regulation, increase or activation; ↓: down-regulation, decrease or inhibition

Astaxanthin (AST) is a xanthophyll carotenoid belonging to the terpenoids class, found in various microorganisms, phytoplankton, marine animals, and seafood [120]. Luo et al. investigated LPS-induced RAW264.7 cells and mice with ALI and discovered that Astaxanthin mitigated inflammatory responses, inhibited ferroptosis, and ameliorated lung damage through the activation of the Keap1-Nrf2/HO-1 pathway [121]. Panax ginseng is a well-known botanical species utilized in traditional medicine for its detoxifying properties, blood glucose regulation, prevention of arteriosclerosis, and potential anti-aging effects [119, 122]. The pharmacological efficacy of ginseng is primarily attributed to its polyacetylene compounds. Panaxydol (PX) is a polyacetylene molecule that has been extensively studied for its diverse biological properties, including anti-fatigue, anti-tumor, and neuroprotective effects [123–125]. In the LPS-induced mouse lung injury model, endotoxin infection increases alveolar capillary permeability, leading to fluid and protein leakage into the alveoli, which causes pulmonary edema and lung tissue damage. These conditions show improvement following PX intervention. Further, PX effectively mitigates LPS-induced ferroptosis in ALI through the Keap1-Nrf2/HO-1 pathway, suggesting its potential as a novel therapeutic option for ALI treatment [30]. Urolithin A (UA) is a secondary metabolite derived from the gut microbiome metabolism of ellagitannins and ellagic acid, which are abundant in pomegranates, strawberries, and various nuts [126, 127]. UA, a phenolic compound, significantly reduced histological alterations, the wet-to-dry lung weight ratio, and the invasion of inflammatory cells, thereby offering protection against LPS-induced ALI in mice. The underlying mechanism involves the activation of the Keap1-Nrf2/HO-1 pathway, which subsequently elevates antioxidant levels in lung tissue and reduces ferroptosis [128]. Obacunone (OB) is a naturally occurring flavonoid commonly found in citrus fruits and is known for its anti-inflammatory and antioxidant properties [129, 130]. Research demonstrated that OB significantly mitigated lung histopathological injury, reduced the release of inflammatory cytokines, and decreased levels of Fe^2+^ and 4-HNE, by inhibiting Nrf2 ubiquitination and upregulating the Nrf2/SLC7A11/GPX4 signaling pathway, ultimately inhibiting iron-dependent ferroptosis and alleviating LPS-induced ALI [131].

Wedelolactone (Wed) is the principal active component of *Eclipta prostrata* and is categorized as a lactone [132]. Research findings indicate that Wed mitigates pancreatitis and associated lung damage in mouse models induced by taurine cholate or small proteins. Specifically, Wed inhibits cell death and ferroptosis in pancreatic and pancreatic acinar cells by upregulating GPX4 [133]. Qingyi decoction (QYD) is a robust anti-inflammatory agent that can improve the intestinal barrier damage caused by SAP, microcirculatory disorders, and pulmonary inflammatory response and has been shown to inhibit both ferroptosis and apoptosis by enhancing the activity of Aldehyde Dehydrogenase 2 (ALDH2). This suggests that QYD has potential therapeutic efficacy in treating lung injury related to severe acute pancreatitis (SAP). Originating from the formula in "Shanghan Lun," as a decoction made from Chinese herbal medicine, QYD is employed in the treatment of acute pancreatitis (AP) patients due to its laxative, heat-clearing, and detoxifying properties [134, 135]. Uncoupling Protein-2 (UCP2) is crucial for managing ROS, maintaining redox balance, and modulating immune responses. Research shows that matrine, an alkaloid from Sophora flavescens, reduces inflammation, oxidative stress, and iron buildup in lung tissue during severe acute pancreatitis-induced acute lung injury (SAP-ALI) by activating the UCP2/SIRT3/PGC1α pathway, highlighting matrine's therapeutic potential for SAP-ALI management [136].

Exposure to PM2.5 has been linked to a multitude of respiratory diseases and was responsible for over 4.2 million deaths in 2015 [137]. Various studies have indicated that PM2.5-induced lung damage is associated with ferroptosis through multiple signaling pathways. One such pathway, the phosphatidylinositol 3-kinase/protein kinase B (PI3K/Akt), is instrumental in regulating the activation of Nrf2, which in turn mitigates lung injury. Sipeimine, a steroidal alkaloid extracted from *Fritillaria roylei*, possesses significant pharmacological attributes, including anti-inflammatory, antitussive, and anti-asthmatic effects [138, 139]. The primary mechanism by which sipeimine ameliorates PM2.5-induced ALI is predominantly through the PI3K/Akt/Nrf2 pathway. This leads to the attenuation of ferroptosis and the restoration of downregulated proteins involved in ferroptosis, such as GPX4, HO-1, and SLC7A11 [140]. Tectoridin, a flavonoid from the rhizome of Belamcanda chinensis, activates the Nrf2 signaling pathway to prevent ferroptosis in lung damage [141]. Similarly, rosavin, a key glycoside from Rhodiola plants, protects against PM2.5-induced lung injury by activating the PI3K/Akt/Nrf2 pathway to inhibit ferroptosis [142]. Astragaloside IV (Ast-IV), a principal glycosidic molecule found in astragalus, effectively modulates ferroptosis and curbs iron-dependent ROS buildup in ALI triggered by PM2.5. It accomplishes this by specifically targeting the Nrf2/SLC7A11/GPX4 axis, showcasing a strategic approach to mitigating the impact of ALI through ferroptosis regulation [143].

ALI resulting from lung or intestinal I/R injury has garnered increasing scholarly attention. This condition is primarily linked to oxidative stress, inflammatory responses, and various modes of cell death, including the recently identified ferroptosis [144]. Ferroptosis, as a novel cell death mode, has been found to be involved in the development of ALI caused by intestinal I/R [145]. Hypoxia-inducible factor (HIF) is a dimeric protein complex, with HIF-1α serving as its main active component. HIF-1α targets HO-1, thereby increasing heme metabolism and subsequently elevating free iron levels, which in turn triggers ferroptosis [146, 147]. Recent research has demonstrated that isoliquiritin apioside (IA), a natural flavonoid derived from *Glycyrrhizae radix et rhizoma*, exerts a protective effect against ALI induced by intestinal I/R. This protective effect is mediated through a HIF-1α dependent mechanism that inhibits ferroptosis in lung epithelial cells [148]. In a study conducted by Zhou et al., IA was found to inhibit the overexpression of HIF-1α and HO-1 proteins, both in vivo and in vitro. Furthermore, when IA was administered to hypoxia/regeneration (H/R)-induced MLE-2 cells, activation of HIF-1α led to increased levels of Ptgs2 and ACSL4, while suppressing GPX4, which are pivotal in initiating ferroptosis [148].

Hyperoxia-induced acute lung injury (HALI) is a life-threatening condition characterized by extensive immune cell infiltration and subsequent apoptosis of type II alveolar epithelial cells (AECII). Salidroside, a bioactive glycoside derived from *Rhodiola rosea*, has been studied for its potential therapeutic effects on HALI. Interleukin (IL)-17A, a critical pro-inflammatory cytokine primarily produced by Th17 cells, is implicated in various diseases, including autoimmune disorders [149] and ALI [150]. Recent research has indicated that salidroside mitigates HALI through IL-17A-mediated ferroptosis. In salidroside-treated HALI models, levels of pro-inflammatory factors such as IL-6, TGF-β, IL-17A, and IL-17RA were found to be reduced. Additionally, the concentration of the ferroptosis biomarker, ferrous ion, was decreased, while the expression of GPX4, a key enzyme in preventing ferroptosis, was elevated [151]. P38 MAPK is a key signaling molecule implicated in both inflammation and ferroptosis [152, 153]. Act1/TRAF6 is a conventional signaling pathway responsible for IL-17A activation and also serves as an upstream signal for p38 MAPK [154]. Further validation in a hyperoxia-induced KM mouse model revealed that salidroside alleviates HALI-associated inflammation and ferroptosis by inhibiting the Act1/TRAF6/p38 MAPK pathway [151].

According to existing data, the incidence of ALI in patients with sepsis is estimated to be over 40%, and a significant percentage of these cases may progress to ARDS [3, 155]. Ferulic acid (FA), a phenolic compound naturally found in various plants and vegetables, has been shown to have therapeutic potential in this context. Studies indicate that ferulic acid can reduce the lung injury score by 48%, inhibit alveolar epithelial cell ferroptosis, and enhance alveolar epithelial barrier function through the activation of the Nrf2/HO-1 signaling pathway in sepsis-induced ALI [156]. Puerarin, a flavonoid monomer, has also been investigated for its protective effects against pulmonary damage in sepsis. Specifically, puerarin was found to suppress both ferroptosis and the inflammatory burst in lung damage induced by sepsis. This was achieved in LPS-induced A549 cells by activating the SLC7A11/GPX4 axis and upregulating FTH1 expression [157]. Paraquat (PQ) poisoning is known to induce ALI and PF, both of which are associated with high mortality rates and limited therapeutic options [158]. Research indicates that the progressive inflammatory responses and lung fibrosis resulting from PQ poisoning are linked to excessive production of ROS through redox cycling [159]. Tripterygium wilfordii Hook.f. (TwHF), a member of the celastraceae family commonly known as lei gong teng in China, primarily contains terpenoids as its active substances [160]. Studies have demonstrated the potential efficacy of TwHF in treating PQ-induced lung fibrosis. Further investigations revealed that ferroptosis plays a role in the pathogenesis of PQ poisoning, and TwHF treatment was shown to inhibit the progression of pulmonary ferroptosis via modulation of the Keap1/Nrf2/HO-1 pathway [161]. Proanthocyanidins (PAs), a class of bioactive flavonoids derived from *Carthamus tinctorius* L, have been shown to protect against ALI induced by Influenza A virus (IAV) and H1N1 through the inhibition of the TGF-β1/Smad signaling pathway. Moreover, PAs were found to suppress IFN-γ-induced ferroptosis, leading to the amelioration of ALI. This was evidenced by a reduction in MDA and ACSL4 levels, along with an upregulation of GSH, GPX4, and SLC7A11 expression [162]. Naringenin, a flavonoid primarily found in fruits like grapefruit and oranges, as well as in vegetables, possesses a range of bioactive properties, including anti-cancer, anti-inflammatory, antioxidant, anti-proliferative, anti-atherosclerotic, and anti-ferroptotic effects [163]. Extensive research has shown that naringenin protects against silver nanoparticles (AgNPs)-induced pulmonary damage by upregulating the Nrf2/HO-1 signaling pathway [164].

To summarize, these studies collectively demonstrate that 7 natural products extracted from herbs, including flavonoids, phenols, alkaloids, terpenoids, polyacetylenes, glycosides, and lactones, with their 17 active ingredients, inhibit ALI caused by a variety of factors, including LPS, IAV, AgNPs, PQ, hyperoxia, intestinal I/R, SAP, and PM2.5. These natural products predominantly modulate the alveolar capillary permeability within the lung tissue, mitigate damage to alveolar epithelial and pulmonary capillary endothelial cells, alleviate pulmonary edema, and attenuate inflammatory responses and oxidative stress. Furthermore, they inhibit lipid peroxidation, curtail iron accumulation, and suppress the induction of ferroptosis, contributing to an enhanced pulmonary function and structural integrity. They primarily regulate the Nrf2/HO-1 and SLC7A11/GPX4 signaling pathways. Additionally, they also affect related signaling pathways, including PI3K/AKT, UCP2/SIRT3/PGC1α, Act/TRAF6/P38MAPK, and TGF-β/Smad. These pathways additionally regulate crucial targets of ferroptosis, including GPX4, ACSL4, SLC7A11, FTH1, FTL, and TER, which ultimately inhibit ferroptosis and ameliorate ALI.

### Chronic obstructive pulmonary disease

COPD, featured with chronic airway inflammation and airflow limitation often linked to smoke, dust, and toxic fumes [165], ranks as the fourth leading cause of death worldwide, yet current treatments are not fully effective [166]. Research indicates that cigarette smoke (CS) exacerbates COPD progression by promoting excessive cellular iron accumulation, facilitating ferroptosis [167]. The use of ferroptosis inhibitors, such as deferoxamine and ferrostatin-1 (Fer-1), shows promise in countering CS-induced ferroptosis in bronchial epithelial cells [168], highlighting an area needing further exploration. Among natural products, curcumin (CUR), a polyphenolic compound from turmeric, has been shown to alleviate ferroptosis by upregulating of the SLC7A11/GPX4 axis and FTH1, downregulating TFR expression, thereby ameliorating lung epithelial cell injury and inflammation induced by CS [165]. Dihydroquercetin (DHQ), a flavonoid, has shown potential in mitigating ferroptosis in COPD by modulating iron transport and activating Nrf2-dependent pathways [17]. Wang et al. reported that the combination of Tongxinluo (TXL) and atorvastatin elevated the levels of GPX4 and ferroptosis suppressor protein 1 (FSP1), while reducing the levels of ACSL4. This led to a decrease in LPO and other key ferroptotic processes by modulating unsaturated fatty acid metabolism, thereby offering a therapeutic approach for COPD complicated with atherosclerosis [31]. Scutellarein is a flavonoid compound derived from plants such as *Scutellaria altissima L*, *S.baicalensis Georgi*, *S. Barbara D. Don*. Liu et al. found that scutellarin prevented RSL3-induced ferroptosis and mitochondrial damage in BEAS-2B cells and alleviated LPS/CS-induced COPD in mice. Mechanistically, scutellarein directly chelates Fe^2+^ and interacts with ALOX15 to reduce lipid peroxidation, reverse GPX4 downregulation, and block Nrf2/HO-1 and JNK/p38 pathway overactivation [169]. According to existing research, the main effect of this intervention is the suppression of ferroptosis, leading to enhanced mitigation of chronic inflammation and airway constriction in individuals with COPD. This is realized through the up-regulation of the SLC7A11/GPX4 axis and FTH1, coupled with the down-regulation of TFR1, and the attenuation of the Nrf2/HO-1 pathway, as delineated in Table 3. The exploration of natural products with ferroptosis-inhibitory properties presents a novel avenue for the development of new therapeutics for COPD.

**Table 3:** Natural products targeting ferroptosis in other pulmonary disease

Disease	Component	Classification	Main roots	Test models	Dose	Mechanisms	Specific effects	Refs.
COPD	Curcumin	Phenols	Turmeric	BEAS-2B cells; Sprague–Dawley male rats	In vitro: 5, 10, 20 μM; In vivo: 100 mg/kg	Up-regulating SLC7A11/GPX4 axis and FTH1; down-regulating TFR1	Up-regulating the protein levels of SLC7A11, GPX4, and FTH1; down-regulating the protein levels of TFR1; decreasing lipid peroxidation, GSH depletion, and iron overload; anti-oxidative stress: decreasing the contents of MDA and ROS	[165]
COPD	Dihydroquercetin	Flavonoids	Onion, French maritime pine bark, milk thistle, and Douglas fir bark	HBE cells;	In vitro: 40, 80 μM; In vivo: 50 mg/kg (low-dose), 100 mg/kg (high-dose)	Activating Nrf2-mediated pathway	Decreasing production of MDA and ROS, increasing SOD activity; up-regulating the protein levels of SLC7A11 and GPX4; decreasing lipid peroxidation; attenuating mitochondria damage	[17]
COPD	Tongxinluo	Formulas	Chinese herbal medicine	HPMECs; male C57BL/6 and ApoE-/- mice	In vitro: 200, 400, 800 μg/ml; In vivo: 0.75 g/kg	Up-regulating the protein expression of GPX4 and FSP1	Increasing protein levels of GPX4, FSP1; decreasing protein levels of ACSL4; ameliorating pathological lung injury and pulmonary function: FRC, RI, Cdyn, MV; ameliorating dyslipidaemia and atherosclerotic lesions; protecting pulmonary microvascular endothelial barrier; enhancing the antioxidant capacity: GSH, SOD, MDA, NO; increasing HPMECs viability	[31]
COPD	Scutellarein	Flavonoids	Scutellaria altissima L; S.baicalensis Georgi; S Barbara D. Don	BEAS-2B cells; C57BL/6 mice	In vitro: 5 μM; In vivo: 5, 10, 20 mg/kg	Chelating Fe2 + and interacting with ALOX15	Chelates Fe2 + and interacts with ALOX15 to reduce lipid peroxidation, reverse GPX4 downregulation, and block Nrf2/HO-1 and JNK/p38 pathway overactivation	[169]
Asthma	FAGS and CK	Other	Ginseng sprouts and its ginsenoside	Female C57BL/6 mice	In vivo: 300 mg/kg (FAGS: low-dose), 600 mg/kg (FAGS: high-dose); 50 μM (CK);	Up-regulating SLC7A11/GPX4 axis	Inducing airway hyperresponsiveness and IgE production; decreasing airway Inflammation: declining contents of inflammatory cells and Th2 cytokines; attenuating oxidative stress: decreasing contents of ROS and MDA; increasing the SLC7A11 and GPX4 expression levels, decreasing the 4-HNE expression level and iron accumulation	[173]
Asthma	Quercetin	Flavonoids	Variety of plants	RAW 264.7 cells; male C57BL/6 mice	In vitro: 10 μM; In vivo: 25 mg/kg	Inhibiting M1 macrophage polarization	Up-regulating expression of SLC7A11 and GPX4; decreasing total levels of inflammatory cytokines: TNF-α, IL-6, IL-1β, and IL-17A; alleviating lipid peroxidation: MDA, 4-HNE; decreasing the mRNA levels of M1'makers: CD86, iNOS, MF1	[174]
PF	Dihydroquercetin	Flavonoids	Yew, larch and cedrus brevifolia bark	HBE cells, MRC-5 cells; C57BL/6 mice	In vitro: 40 μM; In vivo: 10 mg/kg (low-dose), 50 mg/kg (high-dose)	Inhibiting ferritinophagy	Reducing the levels of profibrotic markers: α-SMA, collagen1 and fibronectin; decreasing the levels of ferropotosis relative factors: Fe2 + , ROS, MDA, 4-HNE content, lipid peroxidation; increasing levels of GPX4, GSH; up-regulating the ferritinophagy markers FTH1 and NCOA4, down-regulating autophagy makers LC3	[177]
PF	Tuberostemonine	Alkaloids	Stemona	HLF cells; C57BL/6 mice	In vitro: 350, 550, 750 μM; In vivo: 50 mg/kg (low-dose), 100 mg/kg (high-dose)	Up-regulating SLC7A11/GPX4 axis	Reducing inflammation and collagen deposition; up-regulating SLC7A11, GPX4 and GSH; down-regulating the accumulation of iron and ROS	[178]
LIRI	Tanshinone IIA	Quinones	Salvia miltiorrhiza	C57BL/6 mice	In vivo: 30 μg/kg	Activating the PI3K/Akt/mTOR pathway	Decreasing lung injury score, W/D ratio, MPO and MDA contents; inhibiting inflammatory response: decreasing the expression of IL-1β, IL-6 and TNF-a, increasing the expression of IL-10; inhibiting ferroptosis: increasing levels of GPX4, SLC7A11 and GSH, and decreasing levels of Ptgs2 and MDA; decreasing apoptosis: increasing in the Bcl-2, and decreasing in the Bax, Bim, Bad and caspase3	[187]
LIRI	Salidroside	Glycosides	Rhodiola rosea	MLE-12 cells and RAW 264.7 cells; Male C57BL/6 and Nrf2 − / − mice	In vitro: 40 µM; In vivo: 50 mg/kg	Activating the Nrf2/SLC7A11 signaling axis	Reducing lipid peroxides and iron overload, up-regulating the expression of ferroptosis tightly related proteins Nrf2, SLC7A11, and GPX4	[188]

### Asthma

Asthma is a chronic respiratory disease characterized by persistent inflammation in the airways, leading to symptoms such as wheezing, coughing, chest tightness, and shortness of breath [170]. It is estimated that approximately 300 million people worldwide suffer from asthma, with projections suggesting an additional 100 million will be affected by 2025 [171]. At present, asthma treatment mainly uses bronchodilators, hormones, and theophylline. During the acute attack period, hormone drugs such as albuterol bronchodilator, aminophylline, and prednisone can be used to relieve airway spasm. During the remission period, guidelines recommend that LABA or SABA combined with ICS can be used to improve symptoms and reduce the number of attacks [172]. Ryu et al. demonstrated that fermented and aged ginseng sprouts (FAGS) and compound K (CK) ameliorated various asthmatic markers, including Th2 cytokine production, IgE synthesis, mast cell activation, goblet cell hyperplasia, airway hyperresponsiveness, and inflammation, in a mouse model of allergic asthma. These effects were attributed to the inhibition of inflammatory responses and ferroptosis [173]. Quercetin (QCT), a widely occurring natural flavonoid, has been shown to possess anti-inflammatory and ferroptosis-inhibitory properties across various pathological conditions. In vitro studies have revealed that QCT mitigates LPS-induced ferroptosis by enhancing cell viability and upregulating the expression of antioxidant proteins involved in ferroptosis, specifically SLC7A11 and GPX4. Moreover, in the context of neutrophilic asthma-associated airway inflammation, ferroptosis was observed in conjunction with an elevated M1 phenotype. QCT was found to suppress ferroptosis in both cellular and animal models by inhibiting the pro-inflammatory M1 profile [174]. In conclusion, the current approach to mitigating ferroptosis in asthma mostly involves the inhibition of M1 polarization and inflammation. The key targets for regulating ferroptosis are SLC7A11 and GPX4 (see Table 3).

### Pulmonary fibrosis

PF is a chronic progressive interstitial lung disease characterized by myofibroblast proliferation [175]. The pathogenesis of PF involves both adaptive and innate immune responses, inflammation, injury to epithelial and endothelial cells, epithelial-mesenchymal transition (EMT), and apoptosis [73]. Currently, the clinical treatment of pulmonary fibrosis primarily involves the use of glucocorticoids, immunosuppressants, anti-fibrotic drugs, lung transplantation, and palliative care [176]. However, these treatments do not stop the progression of the disease and do not offer a cure, highlighting the need for the development of new drugs that are safer and more effective. Recent studies have indicated that ferroptosis in lung tissue contributes to the development of PF. Notably, several natural products have demonstrated protective effects against PF. Dihydroquercetin (DHQ), a flavonoid compound, has been shown to inhibit ferroptosis and ameliorate inflammation and silica-induced PF in mice. Further in vitro studies corroborate the protective effect of DHQ, indicating its role in attenuating silica-induced PF by impeding ferritinophagy-induced ferroptosis in human bronchial epithelial cells (HBECs). This effect is characterized by the activation of NCOA4, downregulation of microtubule-associated protein 1A/1B-light chain 3 (LC3), and upregulation of FTH1 [177]. Tuberostemonine, an alkaloid derived from *Stemona*, exhibits inhibitory effects on ferroptosis in a model of bleomycin-induced PF in mice. This inhibition is associated with the upregulation of SLC7A11, GPX4, and GSH and the reduction of iron accumulation and ROS [178]. From the outlined studies, it is evident that natural products mitigate ferroptosis predominantly by modulating ferritin autophagy and the SLC7A11/GPX4 axis, contributing to the amelioration of PF (see Table 3). To elucidate the therapeutic potential of natural products in treating PF, further investigations, encompassing both clinical evaluations and foundational research, are essential, with a particular emphasis on elucidating the role of ferroptosis in this context.

### Lung ischemia–reperfusion injury

Lung ischemia–reperfusion injury (LIRI) is a pathological condition that occurs when the lungs experience a period of insufficient oxygen supply followed by reperfusion [179]. This condition, which can develop after lung transplantation or ischemia in distant organs [180]. LIRI typically manifests in various clinical scenarios, such as cardiac arrest, shock, trauma, pulmonary thrombosis, lung transplantation, and extracorporeal circulation surgery [181]. During LIRI, a surge in reactive oxygen species and pro-inflammatory cytokines can occur, damaging alveolar epithelial cells and the endothelial barrier, leading to pulmonary edema and impaired alveolar gas exchange [182, 183]. New evidence suggests that tissue/cell damage caused by ischemia–reperfusion involves oxidative stress [184] and ferroptosis [185]. Given the high mortality rate associated with LIRI and the lack of effective treatment strategies, there is an urgent need to develop new drugs that can mitigate the pathological features of LIRI [186]. Tanshinone IIA (Tan IIA), an active compound in Salvia miltiorrhiza and a type of quinone [186], has been studied recently. Rui Zhang's research demonstrated that Tan IIA significantly inhibited the decrease in GPX4, SLC7A11, and GSH levels and the increase in Ptgs2 and MDA expression induced by I/R in mice, suggesting that Tan IIA can ameliorate lung ferroptosis caused by I/R injury. The study also utilized LY294002, a PI3K/Akt inhibitor, to further investigate this effect, finding that LY294002 reversed the ferroptosis-inhibitory effect of Tan IIA [187]. Salidroside, a glycoside derived from Rhodiola rosea, exhibits anti-inflammatory and antioxidant properties. Research shows that salidroside effectively reduces LPO and iron overload while enhancing the expression of ferroptosis-related proteins Nrf2, SLC7A11, and GPX4 in mice with LIRI. Additional studies using Nrf2 knockout mice and lung epithelial cell models have confirmed salidroside's ability to inhibit ferroptosis, thereby ameliorating LIRI [188]. According to existing research, the Nrf2/SLC7A11/GPX4 axis is involved in regulating ferroptosis in LIRI (Table 3).

### Pulmonary hypertension

Pulmonary hypertension (PH) is a clinical and pathophysiological syndrome characterized by changes in pulmonary vascular structure or function, leading to increased pulmonary vascular resistance and pulmonary arterial pressure. The global prevalence of PH is estimated at approximately 1% and may rise to over 10% in individuals aged 65 years and above [189]. Currently, the primary clinical treatments for PH include basic therapy, specific treatments, surgical interventions, and targeted combination therapy [190]. However, there are relatively few studies on the role of ferroptosis in pulmonary hypertension. Monocrotaline (MCT), an alkaloid derived from Crotalaria pallida Ait, is an inducer of pulmonary hypertension that closely resembles human PH [191]. Research by Lan’s team found that MCT can induce ferroptosis in pulmonary artery endothelial cells (PAECs), and the use of ferroptosis inhibitors significantly reverses this effect [192, 193]. Astragaloside IV, a natural product, obstructs monocrotaline -induced pulmonary arterial hypertension by improving inflammation and pulmonary artery remodeling [194], but its specific mechanism of action remains unclear. Notably, studies have shown that Astragaloside IV can modulate ferroptosis and alleviate various diseases. It regulates the ferroptosis signaling pathway via the Nrf2/SLC7A11/GPX4 axis, thereby inhibiting PM2.5-mediated lung injury in mice [143]. Astragaloside IV also mitigates cerebral ischemia–reperfusion injury through inhibition of the P62/Keap1/Nrf2 pathway-mediated ferroptosis [195]. Additionally, grape seed proanthocyanidin reduces inflammation and reverses pulmonary vascular remodeling in monocrotaline-induced pulmonary arterial hypertension [196, 197]. Such discoveries provide a theoretical basis and new perspectives for researchers to explore the treatment of pulmonary hypertension with natural products by regulating ferroptosis.

## Discussion

Ferroptosis, a unique form of cell death, is implicated in various diseases, yet its molecular intricacies remain partly elusive, highlighting the need for more research. Insights so far are largely derived from basic research, including in vitro and in vivo studies. The variable absorption, effects, and metabolism of natural products in different species pose challenges for their clinical development as drugs. The extensive time required to secure research support and conduct clinical trials further complicates the translation of natural products into therapeutic agents. Modern drug research demands the use of sophisticated techniques such as liquid chromatography-mass spectrometry, metabolomics, pharmacokinetics, and data mining, along with behavioral, toxicological, molecular biology, and genomic testing [198–200], offering both opportunities and obstacles in advancing natural products from the laboratory to the clinic.

According to the FDA data, 70% of the 1562 new drugs approved between 1981 and 2014 have natural origins [201], and about one-third of drugs over the past 20 years are based on natural products and their derivatives [202]. These figures underscore the safety and effectiveness of natural products, though their toxic side effects and interactions still require careful consideration. For example, studies have shown that curcumin exhibits cytotoxic effects in vascular smooth muscle cells (VSMCs), inhibiting cell proliferation at a dosage of 5 μM and inducing cell senescence and apoptosis when the dosage exceeds 5 μΜ [203]. However, a phase IIa open-label randomized controlled trial with colorectal cancer patients receiving 2 g of curcumin orally daily for 6 months reported no significant side effects, highlighting curcumin's clinical safety [204]. Similarly, a phase 1 study found Atuna racemosa extract effective against methicillin-resistant Staphylococcus aureus (MRSA) with minimal side effects [205]. In contrast, camptothecin and its derivatives, while effective against tumors, have multiple side effects like diarrhea, fatigue, bone marrow suppression, and nausea [206, 207], necessitating methods to mitigate these effects. The interaction of natural products, including synergistic and antagonistic effects, can alter pharmacological activity and side effects [208, 209]. This concept aligns with the seven-emotion compatibility theory in TCM. The development of TCM and modern medicine is interactive and integrated, yet studies on interactions between natural products are limited, urging researchers to employ multidisciplinary and modern scientific approaches. In summary, while natural products have generally been shown to be safe and tolerable in various studies and clinical trials, the safety and side effects of natural products are issues that require thorough research and careful consideration.

Clinical research on targeting ferroptosis in disease treatment is limited, with ongoing studies primarily in clinical trials. A notable double-blind trial demonstrated that curcumin or nanocurcumin significantly outperformed a placebo in anti-inflammatory and antioxidant effects [210]. Similarly, a study on sepsis revealed curcumin's ability to decrease serum inflammatory markers (IL-6, IL-18), oxidative stress indicator MDA, and increase antioxidants like Nrf2, catalase, and SOD, implicating its potential to target ferroptosis-related lipid peroxidation in clinical settings [211]. Between May 22, 2015, and March 12, 2018, in a randomized, open-label, noninferiority phase 3 trial, Nanoparticle albumin-bound (nab-)paclitaxel (100 mg/m2) on days 1, 8, and 15 of a 21-day cycle, were applied for patients with advanced NSCLC previously treated with cytotoxic chemotherapy. After nab-paclitaxel intervention, the patient's immune response and toxic side effects were reduced, the overall survival rate was increased, and the activity against solid tumors was enhanced [212]. Additionally, research on ferroptosis-related lincRNA for lung adenocarcinoma risk stratification suggests new avenues for treating drug-resistant lung cancer [213]. Further validation in larger populations is needed to confirm these findings' generalizability and stability. Combining natural products with nano-delivery systems and ferroptosis-related lincRNA opens a new way to treat drug-resistant patients, especially lung cancer in the future.

In recent years, many ferroptosis-related inducers and inhibitors have been identified, such as ML162, RSL3, FIN56, and FINO2, which are GPX4 inhibitors. These agents contribute to a reduction in GSH synthesis and an increase in lipid peroxidation [214]. Natural products as ferroptosis inhibitors or inducers are a potential area for clinical drug development. For instance, bufotalin, a natural small molecule, acts as a promising GPX4 inhibitor, facilitating GPX4 ubiquitination and increasing intracellular Fe^2+^ levels and lipid peroxidation, thereby promoting ferroptosis and inhibiting lung cancer. Curcumin, conversely, acts in opposition to the ferroptosis inhibitor Fer-1, promoting ferroptosis and inhibiting the proliferation of A549 and H1299 cells [108]. These studies open up new avenues for research, specifically in identifying natural compounds from TCM as potential ferroptosis inducers or inhibitors. Another example is erastin, a well-known inducer of ferroptosis; resveratrol has been reported to inhibit erastin-induced ferroptosis via the Nrf2/Keap1 pathway [112]. The Nrf2/Keap1 signaling pathway is a crucial anti-inflammatory and antioxidant pathway, targeted by many natural products [215]. Dimethyl fumarate, initially used to treat psoriasis, was later discovered to be an inducer of the Nrf2/Keap1 pathway. Subsequent studies have employed it in treating multiple sclerosis, autism, and other multi-system diseases [216, 217]. Since most respiratory diseases involve inflammation and oxidative reactions, developing more natural products as Nrf2/Keap1 pathway inducers for lung disease treatment is promising. This approach offers a new perspective for future research on natural products in treating respiratory diseases. We anticipate the discovery of more such inducers or inhibitors that act on multiple targets and pathways to achieve therapeutic effects.

Epigenetic modifications and post-translational modifications (PTM) are crucial regulatory mechanisms in cellular development and function, influencing gene expression and protein behavior without altering the DNA sequence. Epigenetically, the control of p53 has been shown to regulate the expression of SLC7A11, impacting ferroptosis [65]. Ubiquitination, a PTM process, plays a key role in the degradation of proteins, including those related to ferroptosis. Research has identified that natural compounds like Bufotalin and Sanguinarine can induce GPX4 ubiquitination and degradation, promoting ferroptosis and hindering NSCLC progression [32, 81]. Similarly, 6-Gingerol has been found to inhibit USP14 ubiquitination, enhancing ferritin autophagy and ferroptosis to suppress tumor growth [103]. Additionally, phosphorylation, another PTM type, has been reported to increase LIP and ferroptosis sensitivity, slowing lung adenocarcinoma development [218]. Thus, targeting the epigenetic and PTM pathways of ferroptosis using natural products presents a promising strategy for treating lung cancer and potentially other respiratory diseases.

Indeed, current research on the relationship between natural products and ferroptosis, particularly in the context of lung diseases, is limited, leaving a significant scope for exploration, including negative findings. The theories of TCM, such as the balance of yin and yang, the five elements, qi, blood and body fluids, and the concepts of defending qi and nourishing blood, form the bedrock of TCM's approach to diagnosis and treatment. TCM also emphasizes harmony between humans and nature, holistic concepts, syndrome differentiation and treatment, and the rationale behind prescriptions. There is a growing trend of integrating TCM theories with modern scientific approaches. Modern medical experiments and clinical research are increasingly being employed to validate the scientific basis of disease diagnosis and treatment as guided by TCM principles. Moreover, modern technologies like chemical composition analysis, efficacy evaluation, and drug synthesis are crucial in verifying the mechanisms of action, safety, effective dosage, and potential toxic and side effects of Chinese herbal medicines, including their extracts and active ingredients. Guided by the theories of both traditional Chinese and Western medicine, and augmented by technical advancements in natural product screening, extraction methods, molecular modeling, computational chemistry, high-throughput screening (HTS), drug design and chemical synthesis, as well as pharmacokinetics and pharmacodynamics studies (PK/PD), a comprehensive approach is emerging for new drug research and development [208, 219, 220]. However, due to the conceptual and methodological differences between TCM and modern medicine, fully integrating these two approaches necessitates more in-depth research and exploration.

## Conclusions

**Fig. 3:**
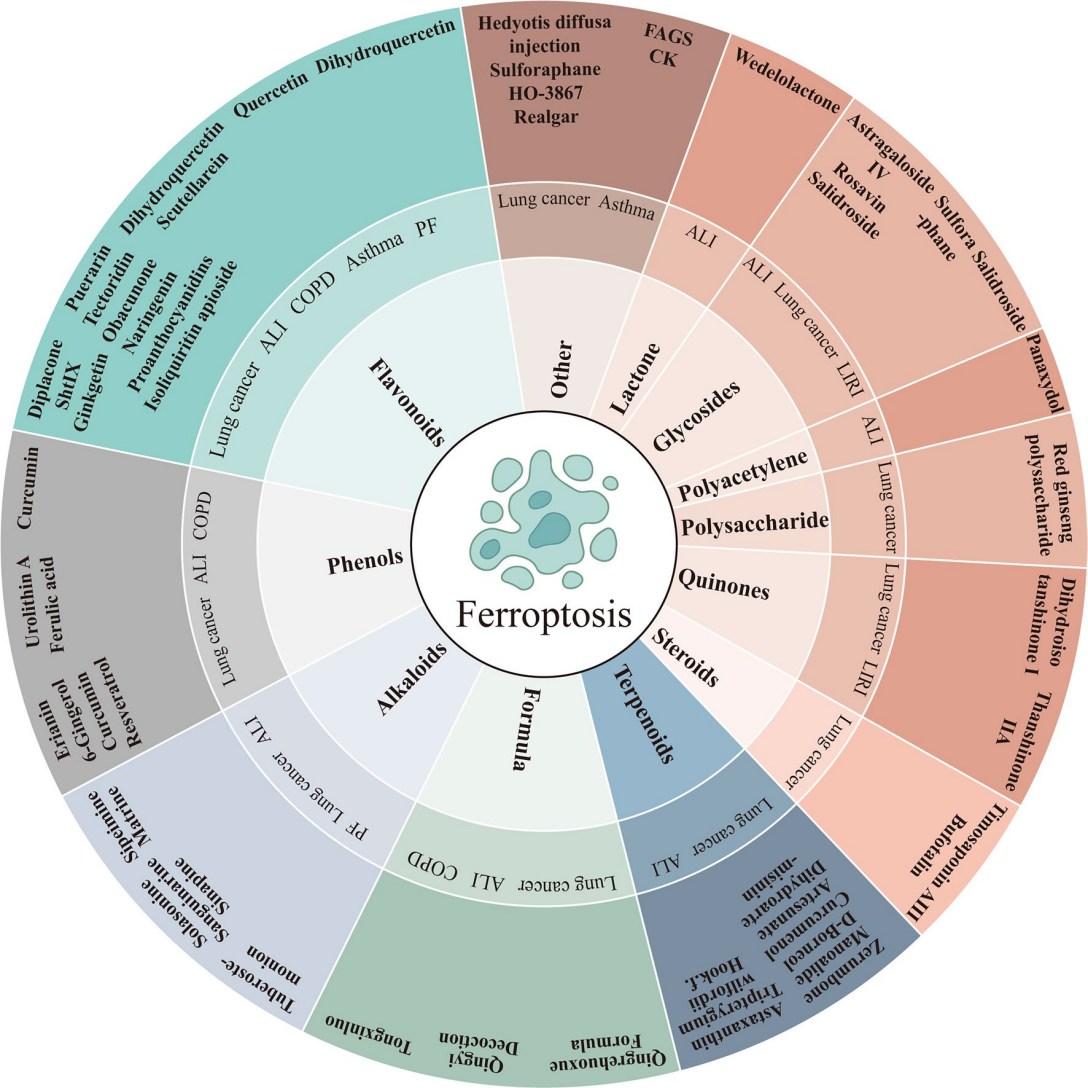
Classification of natural products in targeting ferroptosis in respiratory conditions. Diseases including lung cancer, ALI, COPD, asthma, PF, LIRI and PH. Flavonoids, phenols, alkaloids, terpenoids, steroids, quinones, polysaccharides, polyacetylenes, glycosides, and lactones are 10 categories of natural products, each contributing to the diverse and complex array of biochemical compounds derived from nature

In summary, our review compiles information on 43 natural products that modulate ferroptosis for treating respiratory diseases, including lung cancer, Acute Lung Injury (ALI), Chronic Obstructive Pulmonary Disease (COPD), Pulmonary Fibrosis (PF), asthma, Lung Ischemia–Reperfusion Injury (LIRI), and Pulmonary Hypertension (PH). This compilation primarily encompasses the sources and classifications of these natural products, their principal mechanisms or targets in regulating ferroptosis, and their specific effects. The associated respiratory diseases and classifications of the natural products are illustrated in Fig. 3. Presently, the natural products we have identified that act on ferroptosis are primarily categorized into ten groups: flavonoids, phenols, alkaloids, terpenoids, steroids, quinones, polysaccharides, polyacetylenes, glycosides, and lactones. The field of ferroptosis research is advancing swiftly, presenting both promising opportunities and considerable challenges. This review elucidates factors such as unstable iron accumulation, elevated lipid peroxidation, inhibition of GPX4, impairment of system Xc − , depletion of GSH, ferritin autophagy, the Fenton reaction, and increased ROS are primary contributors to ferroptosis in respiratory diseases. Moreover, key pathways and targets involved include the Keap1/Nrf2/HO-1 signaling pathway, the P53/SLC7A11 axis, the Nrf2/SLC7A11/GPX4 axis, TFR1, NCOA4, ACSL4, VDAC2/3, LVDCC, and USP14. These relevant targets and markers play a pivotal role in mitigating ferroptosis to improve respiratory conditions. The natural products discussed mainly exhibit antioxidant, anti-inflammatory, anti-tumor, and immunomodulatory effects, among others. Some have been shown to act on specific biomolecules like enzymes, receptors, or cell membrane channels, influencing cell signaling, metabolic pathways, or gene expression. Future research should leverage multidisciplinary approaches, including the study of ferroptosis-related biomarkers and signaling pathways, the application of nanotechnology and mitochondrial targeting, and the use of modern drug extraction techniques. The goal is to develop safer and more effective drugs or diagnostic products derived from natural products for the diagnosis, examination, treatment, and prognosis of clinical diseases. While the effects of certain natural products on ferroptosis have been identified, the intricate molecular mechanisms behind many others still require extensive investigation. This review seeks to lay the groundwork for a deeper understanding of the regulation of ferroptosis by natural products in respiratory disease treatment. It is our hope that this contribution will spark further research and provide foundational guidance for the clinical use of TCM in managing various respiratory ailments.

## Data Availability

The data used to support the findings of this study are available from the corresponding author upon request.

## Abbreviations

TCM: Traditional Chinese medicine
PCD: Programmed cell death
ALI: Acute lung injury
PF: Pulmonary fibrosis
COPD: Chronic obstructive pulmonary disease
LIRI: Lung ischemia–reperfusion injury
PH: Pulmonary hypertension
ARDS: Acute respiratory distress syndrome
GSH: Glutathione
GPX4: Glutathione peroxidase 4
ROS: Reactive oxygen species
PAO1: P. aeruginosa
LPO: Lipid peroxide
TF: Transferrin
TFR1: Transferrin receptor 1
TFRC: Transferrin receptor
NCOA4: Nuclear receptor coactivator 4
SLC40A1: Solute carrier family 40 member 1
STEAP3: Six transmembrane epithelial antigen 3
DMT1: Divalent metal transporter 1
FTL: Ferritin light chain
FTH1: Ferritin heavy chain 1
PUFAs: Polyunsaturated fatty acids
ALOX15: Arachidonic acid lipoxygenase 15
ACSL4: Acyl-CoA synthetase long chain member 4
LPCAT3: Lysophosphatidylcholine acyltransferase 3
AA: Arachidonic acid
AdA: Adrenaline
PE: Phosphatidyl ethanolamine
MDA: Malondialdehyde
4-HNE: 4-Hydroxynonenal
L-OOH: Lipid hydroperoxides
L-OH: Lipid alcohols
Glu: Glutamic acid
Cys: Cysteine
Gly: Glycine
System Xc −: Cystine/glutamate antiporter system
SLC7A11: Solute carrier family 7 member 11
SLC3A2: Solute carrier family 3 member 2
PHGPX: Phospholipid hydroperoxide glutathione peroxidase
GSSG: Glutathione disulfide
GCLC: Gamma-cysteine ligase
VDACs: Voltage-dependent anion channels
MMP: Mitochondrial membrane potential
MPTP: Mitochondrial permeability transition pore
Nrf2: Nuclear factor erythroid 2-related factor 2
HO-1: Heme oxygenase-1
NADPH: Nicotinamide adenine dinucleotide phosphate
NQO1: Quinone oxidoreductase 1
SAT1: Spermine/spermidine N1-acetyltransferase 1
NSCLC: Non-small cell lung cancer
SS: Solasonine
CaM: Calmodulin
DP: Diplacone
ATF3: Activating transcription factor 3
QRHXF: Qingrehuoxue Formula
p-GSK-3β: Phospho-glycogen synthase kinase-3
DT: Dihydroisotanshinone I
SAG: Sanguinarine
RGP: Red ginseng polysaccharide
LDH: Lactate dehydrogenase
Tim-AIII: Timosaponin AIII
ShtIX: S-3′-hydroxy-7′, 2′, 4′-trimethoxyisoxane
DDP: Cisplatin
MA: Manoalide
HDI: Hedyotis diffusa injection
PRNP: Prion protein
ART: Artesunate
DHA: Dihydroartemisinin
SFN: Sulforaphane
SI: Sinapine
CUR: Curcumin
USP: Ubiquitin-specific protease
LUSC: Lung squamous carcinoma
MODS: Multiple Organ Dysfunction Syndrome
LPS: Lipopolysaccharide
I/R: Ischemia/reperfusion
PA: Pseudomonas aeruginosa
HIF-1α: Hypoxia-inducible factor 1α
AST: Astaxanthin
PX: Panaxydol
UA: Urolithin A
OB: Obacunone
Wed: Wedelolactone
QYD: Qingyi decoction
ALDH2: Acetaldehyde dehydrogenase 2
SAP-ALI: Severe acute pancreatitis-induced acute lung injury
AP: Acute pancreatitis
UCP2: Uncoupling protein-2
PI3K/Akt: Phosphatidylinositol 3-kinase/protein kinase B
Ast-IV: Astragaloside IV
IA: Isoliquiritin apioside
H/R: Hypoxia/regeneration
HALI: Hyperoxia-induced acute lung injury
AECII: Type II alveolar epithelial cells
IL: Interleukin
MAPK: Mitogen-activated protein kinase
FA: Ferulic acid
PQ: Paraquat
TwHF: Tripterygium wilfordii Hook.f
PAs: Proanthocyanidins
IAV: Influenza A virus
AgNPs: Silver nanoparticles
CS: Cigarette smoke
Fer-1: Ferrostatin-1
DHQ: Dihydroquercetin
TXL: Tongxinluo
FSP1: Ferroptosis supressor protein 1
FAGS: Fermented and aged ginseng sprouts
CK: Compound K
QCT: Quercetin
EMT: Epithelial-mesenchymal transformation
DHQ: Dihydroquercetin
HBECs: Human bronchial epithelial cells
LC3: Light chain 3
Tan IIA: Tanshinone IIA
MCT: Monocrotaline
PAECs: Pulmonary artery endothelial cells
VSMCs: Vascular smooth muscle cells
MRSA: Methicillin-resistant *Staphylococcus* *aureus*
PTM: Post-translational modifications
HTS: High-throughput screening
PK/PD: Pharmacokinetics and pharmacodynamics
